# Optic glioma and precocious puberty in a girl with neurofibromatosis type 1 carrying an R681X mutation of *NF1*: case report and review of the literature

**DOI:** 10.1186/s12902-015-0076-4

**Published:** 2015-12-15

**Authors:** Mirjana Kocova, Elena Kochova, Elena Sukarova-Angelovska

**Affiliations:** Department of Endocrinology and Genetics, University Pediatric Clinic, Vodnjanska 17, 1000 Skopje, Macedonia

**Keywords:** Neurofibromatosis, NF1, Precocious puberty, Optic glioma, R681X

## Abstract

**Background:**

Neurofibromatosis type 1 (NF1) is a common autosomal dominant genetic disorder with an extremely variable phenotype. In childhood NF1 can be associated with optic glioma and central precocious puberty; the latter is more common when the optic chiasm is affected. The mutational spectrum of the NF1 gene is wide and complex; R681X is a rare severe mutation of the NF1 gene known to cause truncation of neurofibromin, with only ten reported cases in the literature so far.

**Case presentation:**

We describe a girl with NF1 associated with early central precocious puberty appearing at 2.5 years of age and optic glioma affecting the optic chiasm as seen on magnetic resonance imaging (MRI). Genetic analysis confirmed the presence of R681X. Therapy with a gonadotropin-releasing hormone agonist was instituted with good response to therapy. The lesions on MRI were stable and no significant vision impairment was present during the 6 years of follow-up.

**Conclusion:**

Of the ten reported cases of NF1 due to R681X, one has presented with optic glioma and none with precocious puberty. Thus, to our knowledge, this is the first reported case of this mutation presenting with precocious puberty. We believe that this is a contribution to the few reports on the phenotype of this mutation and to the future elucidation of genotype-phenotype correlations of this disease.

## Background

Neurofibromatosis type 1 (NF1) is one of the most common autosomal dominant genetic disorders, affecting approximately 1 in 3500 individuals worldwide [1]. The primary clinical features are café-au-lait spots which progress throughout life to benign peripheral nerve sheath tumors or neurofibromas and Lisch nodules (iris hamartomas). However, other complications, such as skeletal dysplasias, learning disabilities, mental retardation, seizures, and optic glioma fall within the wide clinical spectrum of the disease. Characteristic of NF1 is extreme clinical variability, even in familial cases [2–4]. The National Institutes of Health (NIH) provide the well known diagnostic criteria; presence of at least two of these criteria is sufficient for the diagnosis [5].

NF1 is caused by defects in the *NF1* tumor suppressor gene located at chromosome 17q11.2 and spanning across approximately 300 kb of genomic DNA. *NF1* is composed of 60 exons with at least 4 alternatively spliced exons which are expressed in a developmental- and tissue-specific pattern [6–9]. The *NF1* gene encodes neurofibromin, a protein containing 2818 amino acids (AA) which harbors a functional GAP (GTPase-activating protein)-related domain (GRD, AA 1205–1536) in its central region. Neurofibromin is ubiquitously expressed and most abundant in the nervous system. The protein is highly conserved among vertebrates and shows 60 % identity with the *Drosophila* homolog [10–12]. The mutational spectrum of the *NF1* gene is complex due to the large number of coding exons and the considerable mutational heterogeneity. Most of the mutations occur in eight exons/flanking regions, representing only 16 % of the coding region. Mutations that cause skipping of exon 7, 30, and 29 are very common [13, 14]. Most of the mutations result in truncation and loss of function of neurofibromin. No specific genotype/phenotype correlation has been revealed [15].

The most common NF1-associated tumor is the benign peripheral nerve sheath tumor or neurofibroma. In a small percentage of NF1 patients, plexiform neurofibromas progress to malignant peripheral nerve sheath tumors. While defects in the peripheral nervous system glial cells (Schwann cells) underlie neurofibroma development, NF1 patients are also predisposed to astrocytic brain tumors, spinal tumors, pheochromocytoma, myeloid leukemia and gastrointestinal stromal tumors [1, 16]. Between 15 and 50 % of NF1 patients develop some type of glioma; these are often indolent in nature [17, 18]. Optic nerve gliomas occur in 12–15 % of patients with NF1 usually within the first decade of life [1]. No specific mutation of the *NF1* gene has been associated with localization of the glioma at the optic chiasm. Precocious puberty due to optic glioma is not rare in patients with NF1, especially when the optic chiasm is involved [1–4, 19]. In fact, it is among the most common endocrine disorders in these patients and becomes more frequent with long-term follow up [20, 21]. However, very few patients with NF1 associated with optic glioma or precocious puberty have been molecularly characterized.

Here we present a child with NF1 and optic glioma, who presented with precocious puberty at the age of 2.5 years and was found to bear a rare mutation of the *NF1* gene, R681X. We have reviewed the available literature and here we summarize the clinical findings in published case reports with this mutation.

## Case presentation

**Fig. 1:**
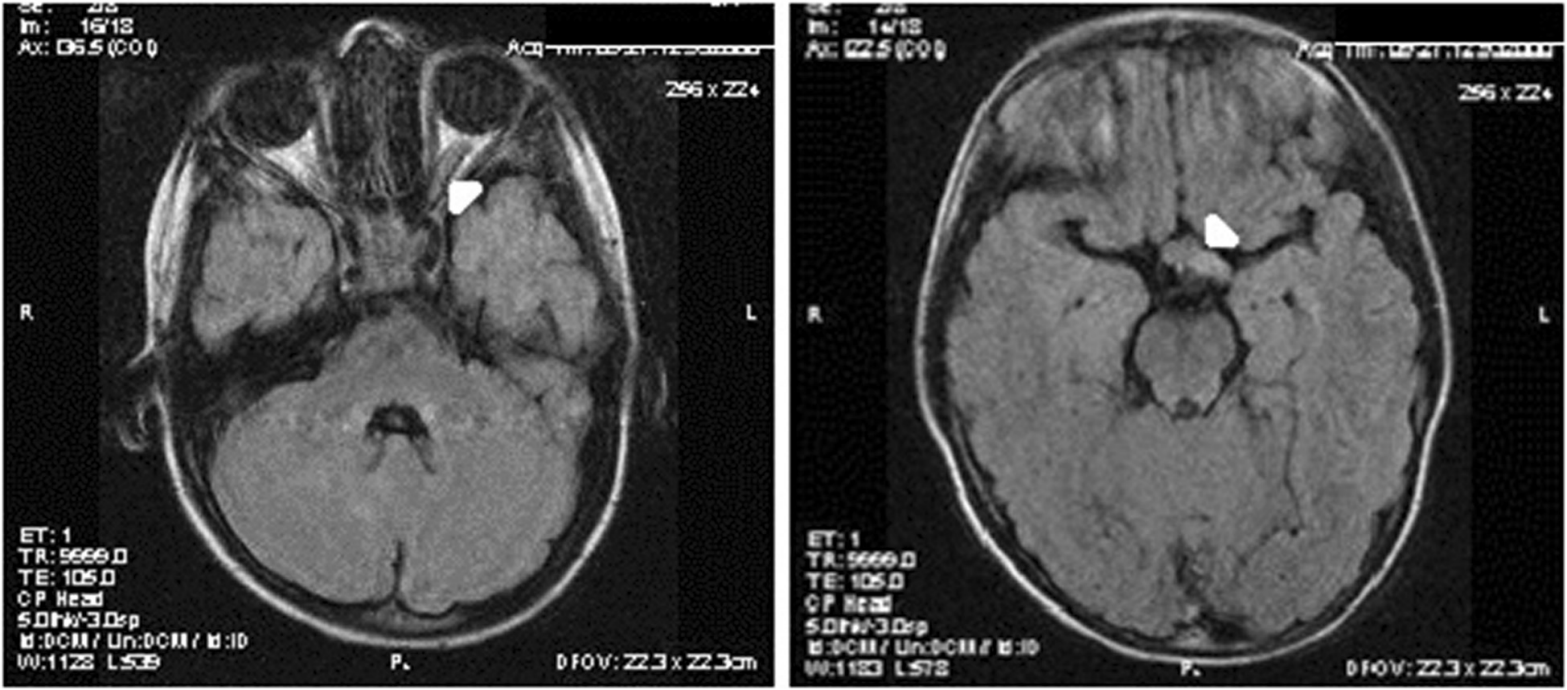
MRI showing optic glioma affecting the optic chiasm; left - thickening of the optic nerve (arrowhead), right - glioma at the optic chiasm (arrowhead)

Our patient was a Caucasian girl born from a normal, well controlled, uneventful pregnancy, delivered by Caesarean with average anthropometric parameters. No family history of neurofibromatosis was reported. At the age of five months, her parents had noticed café-au-lait spots on the skin that were steadily increasing in size over time. At the age of 2.5 years, breast enlargement was noticed and she was referred for hormonal work-up. Her height and weight were at the 95th percentile according to the charts of Tanner and Whitehouse. Telarche was at stage B2. Numerous café-au-lait spots on the skin were noted, various in size, the largest of which measured 30x40 mm. Axillary freckles were present bilaterally. No Lisch nodules or oculomotor difficulties were detected. Fundoscopy revealed pallor of the optic nerves bilaterally. Blood counts were normal. Peak serum values of LH = 16.4 IU/dl and of FSH = 45.3 IU/dl on the GnRH test confirmed the diagnosis of precocious puberty of central origin. The growth hormone level after L-dopa stimulation was normal (peak value 12.3 ng/ml) and was completely supressed with the glucose tolerance test (0.6 ng/ml). The IGF-1 value was within the normal range (289 pg/ml), confirming no GH secretion abnormalities. Magnetic resonance imaging (MRI) of the pituitary region showed a normal pituitary gland with significant thickening of both optic nerves and the optic chiasm (optic glioma). The signal was enhanced by Gadolinium (Table 1, Fig. 1). Molecular analysis of the *NF1* gene showed presence of the R681X mutation.

**Table 1:** Clinical presentation and diagnostic procedures in presented case

Age at diagnosis	2.5 years
Height at diagnosis	+2.5 SDS
Bone age	+1.1 SDS
Puberty	B2, P1
Peak LH value after GnRH stimulation	16.4 IU/dl
GH value after L-Dopa stimulation/glucose tolerance test	12.3 ng/ml / 0.6 ng/ml
IGF-1	289 ng/ml
MRI	Optic glioma spreading to the chiasm
Visual field	Peripheral narrowing of the visual field
Ultrasound of the ovaries	Normal

**Fig. 2:**
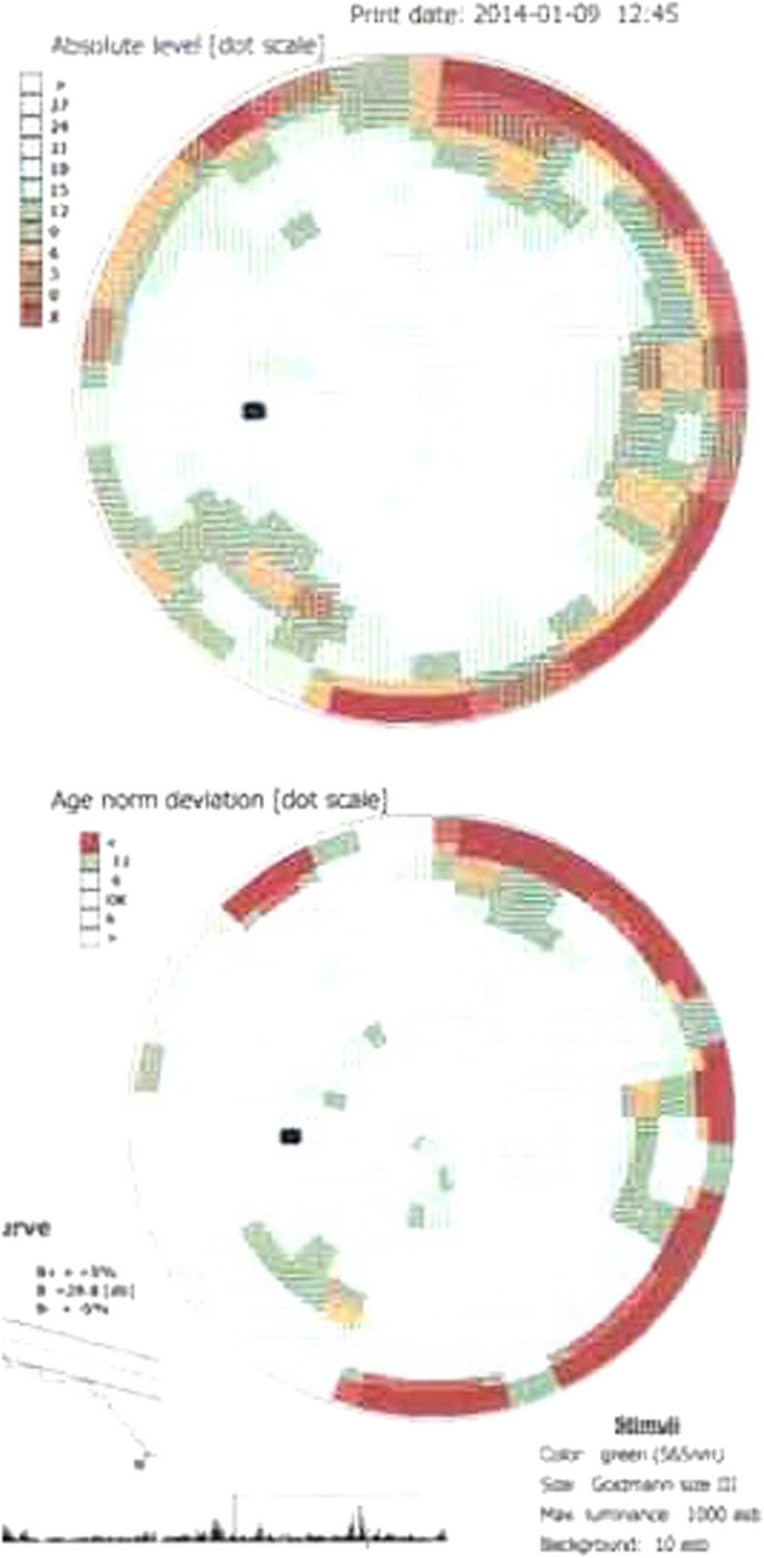
Peripheral visual field affection

Therapy with a GnRH agonist (Triptorelin) at 28-day intervals was instituted and the patient has been receiving this therapy for 6 years thus far. With therapy, growth continued at a normal rate (on the 75th percentile) and the breasts returned to pre-pubertal stage B1. MRI was performed at yearly intervals and did not reveal any enlargement of the optic glioma. The first check-up of the visual field was performed at the age of 6 years and showed minor peripheral loss of vision (Fig. 2).

Although this case initially presented with a complex combination of clinical features, the diagnosis of NF1 was straightforward, due to our significant clinical experience and promptly conducted focused diagnostic procedures, as well as the genetic confirmation of the disease. As such, even though the prognostic characteristics of this particular case are difficult to assess, the long term follow-up of our patient has given some insight into the evolution of the disease.

We searched the Pubmed database using the keywords “neurofibromatosis” “NF1”, “mutation”, “R681X”, and found ten pediatric cases with a reported R618X mutation; all are presented in Table 2. Optic glioma has so far been reported in only one patient with this mutation, however, with no associated precocious puberty [22].

**Table 2:** Phenotype in published cases with confirmed R681X mutation of *NF1*

N^o^ of patients with R681X/Total N^o^ of study participants	Phenotype	Reference	
		Authors	Journal [Reference N^o^]
3/189	Learning disability (2 cases)	Ars E et al.	J Med Genet 2003 [13]
1/16	Optic glioma; Stargardt’s disease	Gerth C et al.	Graefes Arch Clin Exp Ophtalmol 2002 [22]
4/500	?	Fashold M et al.	Am J Hum Genet 2000 [7]
1/108	?	Origone P et al.	Hum Mut 2002 [14]
1/3	Cutaneous neurofibromas	Maertens O et al.	Hum Genet 2007 [32]
1	Optic glioma + Precocious puberty	Kocova M et al.	BMC Endocr Disord

NF1 is caused by mutations in the *NF1* gene, one of the largest human genes bearing one of the highest mutation rates in the human genome. Although most of the described mutations are private, several hot spots with a higher mutation rate such as exons 4b, 7, 10b, 13, 15, 20, 29 and 37 have been described. No strict genotype/phenotype correlations have been confirmed in large studies [13–15, 23]. However, some of the signs of NF1, such as plexiform neurofibroma, scoliosis and learning disabilities have been associated with specific mutations [13].

Association of NF1 with optic glioma or precocious puberty is not rare, but has rarely been molecularly characterized [13, 14, 17–19]. Among 20 patients with optic glioma, *NF1* mutations were detected in 12. Most of these mutations were in the first exons of the *NF1* gene and three of them were located in exon 7; all mutations were different and produced truncated proteins [24]. The R681X (2041C > T) mutation is a nonsense mutation in exon 13 producing a truncated protein composed of 680 amino acids. A familial R681X mutation has been found in only two siblings in one family so far [13]. Central nervous system neoplasms can appear in patients with NF1, the most common being a visual pathway glioma involving one or both optic nerves, the chiasm, or other segments of the visual pathway [21, 25, 26]. These tumors can present clinically with unilateral or bilateral proptosis, decreased vision in one or both eyes, optic nerve pallor or restricted extraocular movements. Pure chiasmatic tumors do not cause proptosis; this was also absent in our patient. Tumors of the optic chiasm are usually associated with a variable degree of loss of visual acuity and visual field unilaterally or bilaterally [27]. However, there is often discrepancy between the tumor size and visual impairment. Many patients will have appropriate vision, but some will be blind [28–30]. In children with so-called asymptomatic optic nerve tumors, careful clinical examination often reveals some degree of optic nerve pallor or restricted ocular movement. Central scotomas, a measurable depression of central vision, occur in approximately 70 % of patients. Peripheral field defects are common, but they are also variable and include quadrantic or hemianopic fields. Bitemporal hemianopic visual field loss occurs in less than one half of patients [29]. Our patient did not have any clinical signs of decreased vision at diagnosis. The ophthalmological examination revealed only a subtle decrease of the peripheral visual field and optic nerve pallor. Long-term follow-up did not show any progression of the optic and chiasmatic glioma, therefore no therapy was indicated. Most of the reported patients in the literature have a similar evolution, i.e. after the age of 6 years no further progression is expected [25, 30].

If the tumor is large enough, hypothalamic dysfunction occurs, and according to some recent data, optic pathway glioma might have stronger causative role for precocious puberty than the presence of NF1 condition [20, 21, 31]. Long-term follow up is necessary since patients with optic glioma and precocious puberty could progress towards gonadotropin deficiency later in life [21].

Reported NF1 patients from the literature carrying the R681X mutation have a variety of clinical presentations (Table 2). The clinical presentation of NF1 associated with optic glioma affecting the optic chiasm together with precocious puberty found in our patient is a combination of clinical features that has thus far never been associated with the R681X mutation.

## Conclusions

The R681X mutation is a very rare mutation of the *NF1* gene. In the literature we found a total of 10 reported cases of NF1 with this mutation so far, presenting with a heterogeneous phenotype. Optic glioma was reported in only one of these cases. The association of this mutation with precocious puberty present in our patient is the very first to our knowledge. The precise influence of this and other mutations of the *NF1* gene on the resulting phenotypic expression remains to be elucidated.

### Consent

All procedures were performed according to the Declaration of Helsinki and approved by the Ethics Committee within the University Pediatric Clinic. Written informed consent was obtained from the patient’s parent for publication of this Case report and any accompanying images. A copy of the written consent is available for review by the Editor of this journal.

### Availability of data

All patient data, including laboratory analyses, imaging scans, and genotyping results are part of the electronic and paper patient record system at the University Pediatric Clinic of Skopje, available upon request.

## Abbreviations

AA: Amino acids
GAP: GTP-ase activating protein
GnRH: Gonadotropin-releasing hormone
GRD: GAP-related domain
MRI: Magnetic resonance imaging
NF1: Neurofibromatosis type 1
*NF1*: Neurofibromin-1 gene
NIH: National Institutes of Health
